# Detection of Salivary Interleukin 2 and Interleukin 6 in Patients With Burning Mouth Syndrome

**DOI:** 10.1155/MI/2006/54632

**Published:** 2006-02-06

**Authors:** Daria Simčić, Sonja Pezelj-Ribarić, Renata Gržić, Jelena Horvat, Gordana Brumini, Miranda Muhvić-Urek

**Affiliations:** School of Dentistry, Medical Faculty, University of Rijeka, 51000 Rijeka, Croatia

## Abstract

The etiology of BMS remains unknown. Role of various cytokines has been implicated in the development of BMS. The aim of this study was to evaluate levels of salivary IL-2 and IL-6 in patients with BMS, compared with age-matched healthy volunteers (control group). Whole saliva from 30 patients with BMS, age range 55–65, was tested for the presence of IL-6 and IL-2 by enzyme immunoassay. Control group consisted of 30 healthy participants, aged 55–65 years. Saliva IL-2 concentrations in BMS were significantly increased in patients compared to healthy subjects: mean 34.1 ± 9.7 versus 7.3 ± 3.0 pg/mL; *P* < .001. Patients with BMS had significantly higher concentrations of IL-6 compared to control: mean 30.8 ± 5.6 versus 5.2 ± 2.8 pg/mL; *P* < .001. In patients with BMS, IL-2 and IL-6 levels in saliva are elevated, correlating with the severity of illness.

## INTRODUCTION

 Burning mouth syndrome (BMS) is characterized by a continuous, painful burning sensation in a clinically normal-appearing oral mucosa. Affected patients often present multiple oral complaints, including burning, dryness, and taste alterations. Burning mouth complaints are reported more often in women, especially after menopause [1]. Typically, patients awaken without pain but they note increasing symptoms throughout the day and into the evening. The etiology of BMS remains unknown, although a number of local, systemic and psychological factors have been proposed as being of etiopathologic importance. Conditions that have been reported in association with burning mouth syndrome include chronic anxiety or depression, various nutritional deficiencies, type 2 diabetes, and changes in salivary function [2]. However, these conditions have not been consistently linked to the syndrome, and their treatment has had little impact on burning mouth symptoms. Recent studies have pointed to dysfunction of several cranial nerves associated with taste sensation as a possible cause of burning mouth syndrome [3]. In more than one half of the patients with burning mouth syndrome, the onset of pain is spontaneous, with no identifiable precipitating factor. Approximately, one third of the patients relate onset time to a dental procedure, recent illness, or medication course (including antibiotic therapy). Regardless of the nature of pain onset, once the oral burning starts, it often persists for many years [4]. The burning sensation often occurs in more than one oral site, with the anterior two thirds of the tongue, the anterior hard palate and the lower lip mucosa being the most frequently involved [5].

As a nonspecific antigen proliferative factor for all T-lymphocyte subpopulations, IL-2 is an immune regulator playing a major role in inflammatory reactions as well as in tumor control. During inflammation, IL-2 stimulates secretion of proinflammatory cytokines such as IL-1, TNF-α, and TNF-β [6].

IL-6 is a pleotropic cytokine that influences the antigen-specific immune responses and inflammatory reactions. Together with IL-1 and TNF-α (which also stimulate IL-6 secretion), it belongs to the group of main proinflammatory cytokines [7].

The causes of BMS and the pathogenic mechanisms behind it are still unknown. The etiology is presumed to be multifactorial, involving an interaction between biological and psychological factors. A considerable number of local, oral factors have been found to relate to BMS, including xerostomia, hyposalivation, taste disturbance, oral candidiasis, temporomandibular functional disorders, and allergic reactions [8].

## SUBJECTS AND METHODS

Thirty patients with BMS were included in this study. Thirty age-matched healthy volunteers served as a control group. Clinical examination was performed according to the standard clinical criteria. After informed consent had been obtained and medical, dental, and social histories collected, each individual expectorated 10 mL of unstimulated whole saliva into a sterile centrifuge tube. Saliva specimens were collected from each participant in sitting position. Afterwards, the saliva specimens were stored at −80°C until the beginning of analysis. For determination of salivary levels of IL-2 and IL-6, ELISA (Sigma Immunochemicals, St Louis, Mo, USA) was performed. The assay was performed according to the manufacturer's instructions, and the results are expressed in pg/mL. The detection limit for both IL-2 and IL-6 was 4.6 pg/mL.

## STATISTICAL ANALYSIS

Data are presented as means with standard deviations, and as median values with interquartile range (IQR) and a logarithmic scale. The results obtained were compared using the nonparametric Wilcoxon test and one-way ANOVA test. Statistically significant difference was defined at *P* < .05.

## RESULTS

**Figure 1:**
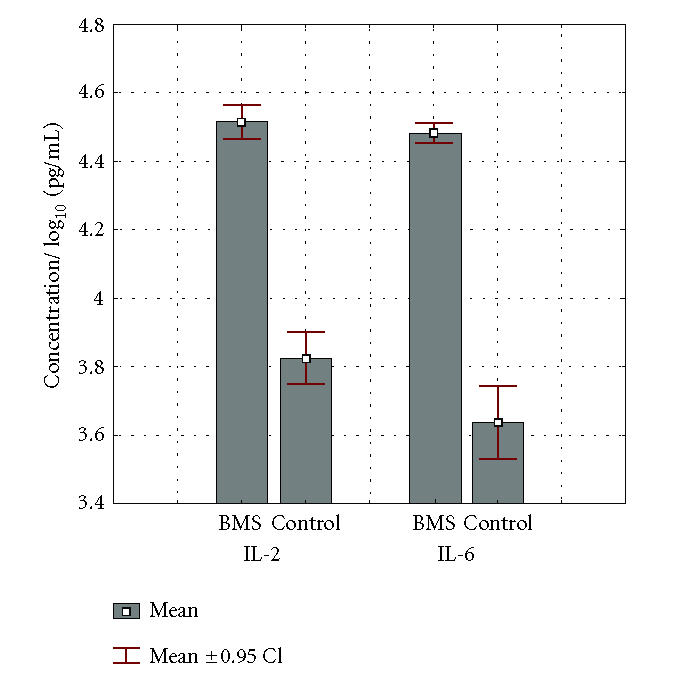
Comparison of IL-2 and IL-6 levels in the patients with BMS and healthy individuals (controls). Data represent the mean log cytokine content (IQR) in pg per milliliter of saliva derived from 30 subjects.

The levels of IL-2 and IL-6 were measured in the whole saliva of control group, as well as in the patients with BMS, and are presented as the median (IQR) on a logarithmic scale in Figure 1.

Concentration of IL-2 was measured in saliva samples of 30 patients suffering from burning mouth syndrome, as well as 30 healthy subjects. Average value of IL-2 concentration was significantly higher in the group of patients with BMS: mean 34.1 ± 9.7 pg/mL compared to control group: mean 7.3 ± 3.0 pg/mL (*P* < .001). Statistical difference was significant at the level of *P* < .001. Median value for IL-2 level in the group with BMS: 34.9 (IQR: 32.5–40.0) pg/mL was significantly higher (*P* < .001), compared to 7.3 (IQR: 5.6–11.4)pg/mL in healthy subjects.

Concentration of IL-6 was determined in saliva specimens of 30 patients suffering from BMS and 30 healthy individuals from control group.

Average value of IL-6 concentration was significantly higher in experimental group: mean 30.8 ± 5.6 pg/mL compared to values obtained in control group: mean 5.2 ± 2.8 pg/mL. The difference was statistically significant at the level of *P* < .001. Median value of IL-6 level in the group of patients with BMS: 30.5 (IQR: 28.4–33.2) pg/mL was significantly higher (*P* < .001) compared to 4.4 (IQR: 2.4–7.0) pg/mL in control group. It can be noted that the highest value of IL-6 in control group was lower than the lowest value of IL-6 in the patients with BMS.

## DISCUSSION

Burning mouth syndrome is characterized by a burning sensation of the tongue or other oral sites, usually in the absence of clinical and laboratory findings. Affected patients often present multiple oral complaints, including burning, dryness, and taste alterations. Pain is one of the common sensations which usually indicate tissue damage as a consequence of various external or internal factors [9]. Nevertheless, persisting pain that does not meet criteria for some other cranial neuralgia or other generally described painful syndrome is described by Headache Society as atypical orofacial pain. Such pain does not follow the usual anatomical pain routes or related physiological mechanisms, cannot be alleviated by cutting off the nerve path, and lacks the identifying neuropathic, extraneural, or central origin [10]. Atypical facial pain, together with stomatodynia (where some authors group BMS), atypical odontalgia, and masticatory muscle and temporomandibular joint (TMJ) disorders, as well as idiopathic dysgeusia form the category of idiopathic orofacial pain [11]. Despite the existence of numerous studies dealing with the problem of atypical painful disorders etiology, clear causative factor or mechanism of this painful condition has not yet been defined. Possible local, systemic, neurological, and psychological factors are repeatedly analyzed [12, 13].

Since there are numerous effects of IL-6 in organisms, and subsequently in orofacial area, its presence and increased concentration in saliva of the patients with BMS confirms the hypothesis of the role of IL-6 in BMS genesis. Depression is a common occurrence in the patients with atypical painful conditions and BMS. Controversy in available literature is whether depression leads to BMS or it is a secondary consequence of chronic painful condition [14]. Statistically significant finding of cytokines in our research, as well as presence of cytokines IL-2 and IL-6 which are analyzed in relation to depression, confirms the existence of relationship between depression and BMS.

Our findings can be correlated to the research conducted by Xia et al [15] who studied levels of IL-2 and IL-6 in sera of the patients with or without depression, suffering from BMS. They reported that there was no statistically significant difference in serum levels of IL-2 and IL-6 in the patients with BMS who also suffered from depression, compared to individuals with BMS and without depression. Their conclusion was that depression does not necessarily influence the serum levels of IL-2 and IL-6, but it is possibly related to BMS. Presence of IL-2 and IL-6 in their patients with BMS corresponds to our findings. The difference between the two is that above-mentioned authors proved the presence of increased serum levels of IL-2 and IL-6, while in our research increased concentrations of IL-2 and IL-6 were also detected in saliva.

Streckfus et al [16] detected increased concentrations of IL-2 and IL-6 in saliva specimens of the patients suffering from Sjögren syndrome. Destructive changes in the glands can therefore be related to immunological and inflammatory responses. Forty six to seventy six percent of the patients with BMS complain of dry mouth [17]. Such connection between BMS and dry mouth could affect accumulation of the pain symptoms; burning sensations; feeding, swallowing, and speech difficulties; as well as negative influence on quality of life. In some patients with BMS who complain of dry mouth, changes in both quality and quantity of saliva can be easily detected [18]. Although the relationship between dry mouth and BMS has already been proved, role of decreased salivary flow, or feeling of dry mouth in the patients suffering from BMS has not been entirely elucidated, or recognized for that matter. This research proved existence of IL-2 and IL-6 in all saliva specimens. In patients with BMS, concentration of these cytokines was increased and statistically significant. This supports the assumption that IL-2 and IL-6 are objective markers for diagnostics and detection of painful condition BMS.

Available literature fails to offer us a single position regarding the cause of BMS development. Authors commonly conclude that in order to assess BMS development, one must take into consideration a variety of causative factors, which are not common for the majority of patients. A certain BMS causative factor in one patient does not necessarily cause burning sensations—pain in another individual suffering from the same problem. Different, multiple activities of IL-2 confirm such hypothesis for BMS development. Increased IL-2 concentration in this research offers a possible explanation for the causative mechanism of BMS through immunological reactions during inflammation.

Due to the fact that BMS is a multicausal disorder, the most rational approach would be to analyze multiple immunologically active substances, since their production and effect depend upon numerous, diverse, and overlapping factors. Future research should be based on combined analyses of immunologically active substances, which might be useful for BMS risk assessment and quality of designated therapy.

Oral fluid being the “mirror of body” is a perfect medium to be explored for health and disease monitoring. The translational applications and opportunities are enormous. The ability to monitor health status, disease onset and progression, and treatment outcome monitoring through noninvasive means is the most desirable goal in the health care promotion and delivery.
